# Molecular circuits for associative learning in single-celled organisms

**DOI:** 10.1098/rsif.2008.0344

**Published:** 2008-10-03

**Authors:** Chrisantha T. Fernando, Anthony M.L. Liekens, Lewis E.H. Bingle, Christian Beck, Thorsten Lenser, Dov J. Stekel, Jonathan E. Rowe

**Affiliations:** 1 Systems Biology Centre, University of BirminghamBirmingham B15 2TT, UK; 2 MRC National Institute for Medical ResearchMill Hill, London NW7 1AA, UK; 3 TU/e Techniche Universiteit Eindhoven5600 MB Eindhoven, The Netherlands; 4 Bio Systems Analysis Group, Friedrich Schiller University JenaJena 07743, Germany; 5 School of Computer Science, University of BirminghamBirmingham B15 2TT, UK

**Keywords:** associative learning, Hebbian learning, single-celled organism, plasmid, synthetic biology

## Abstract

We demonstrate how a single-celled organism could undertake associative learning. Although to date only one previous study has found experimental evidence for such learning, there is no reason in principle why it should not occur. We propose a gene regulatory network that is capable of associative learning between any pre-specified set of chemical signals, in a Hebbian manner, within a single cell. A mathematical model is developed, and simulations show a clear learned response. A preliminary design for implementing this model using plasmids within *Escherichia coli* is presented, along with an alternative approach, based on double-phosphorylated protein kinases.

## 1. Associative learning in a single cell

Associative learning is traditionally thought to be confined to animals with nervous systems (Walters *et al*. 1979; Kandel *et al*. 2000; Fanselow & Poulos 2004). The most famous example is Pavlov's dog, which learned to associate the sound of a bell (the conditioned stimulus) with the smell of food (the unconditioned stimulus) and so salivate when the bell was rung. In multi-cellular organisms, the memory trace is stored as a modification of the connectivity between cells, for example as changes in the synaptic strengths between neurons. However, recent work in systems biology reveals molecular circuits that are rather similar to neural networks (Bray 2003) and logic gates (Buchler *et al*. 2003) within individual cells. Because chemical kinetics is Turing universal (Magnasco 1997) and can therefore at least in principle implement arbitrary neural networks (Hjelmfelt *et al*. 1991) and finite state automata (Hjelmfelt *et al*. 1992), there is no reason for biological mechanisms that sustain associative learning to be confined to neural systems.

Hennessey (1979) showed that the single-celled ciliate *Paramecium caudatum* might possibly be capable of being classically conditioned. A paramecium was successfully trained to exhibit an avoidance response to a conditioned vibration stimulus, using an electric shock as the unconditioned stimulus. This response persisted over the entire lifetime of the paramecium, although no inheritance of this response was studied. Associative sensitization and pseudo-conditioning were not ruled out in the experiment, the repetition of which we would welcome.

Although adaptive sensitization and habituation (non-associative learning) have been demonstrated in bacteria (Yi *et al*. 2000), associative learning within a single lifetime has not. An example of sensitization is the autocatalytic upregulation of *phoA* and *phoB* after prior phosphate limitation, resulting in a stronger response to subsequent phosphate limitation (Hoffer *et al*. 2001). Associative learning is distinct from sensitization because it requires learning a correlation between two different stimuli.

A plausible proof of principle of associative learning in single cells, based on autocatalytic RNA, has been recently proposed (Gandhi *et al*. 2007). The motivation of that paper was to demonstrate an important potential function for regulatory RNA networks. One of the mechanisms uses RNA polymerases to replicate RNA strands capable of reversible ligation and cleavage. Our paper differs in three respects. Firstly, Gandhi *et al*'s mechanism is associative, but, interestingly, it does not use a Hebbian process (Gerstner & Kistler 2002). It is analogous to a hypothetical single symmetric bidirectional synapse, whereas our model is analogous to a single-layer perceptron (Haykin 1998). Secondly, Gandhi *et al*'s motivation is not to devise a system primarily for ease of synthesis using standard molecular biology techniques, although certainly a distinct Hebbian (deoxy-)ribozyme circuit is potentially within the scope of constructability using AND-gates (Stojanovic *et al*. 2002). Thirdly, Ghandi *et al*. (2007) use composite variables consisting of the sum of two species concentrations to represent the responses to learning (e.g. *n*_a_=[A]+[AB]), where *n*_a_ is the variable considered to represent the output of learning, and A and AB are individual chemicals. The outputs of our system are not composite variables, but the concentrations of single transcription factors. Our circuit also exhibits extinction as observed in Pavlovian conditioning. We use realistic units (see table 1), which allow us to predict the time scales over which conditioning would be expected to occur in the laboratory.

A recent paper has described how *in silico* evolution could be used to produce gene regulatory networks (GRNs) capable of ‘predictive behaviour’ (Tagkopoulos *et al*. 2008). The environmental affordance that such a mechanism exploits is the existence of environmental temporal regularities that persist for several bacterial generations, for example in marine ecosystems a temperature change may precede a change in O_2_ concentration, and photon flux may precede temperature changes. In the gut, the need to switch from aerobic to anaerobic respiration may be signalled by increasing temperature. In Tagkopoulos *et al*'s task, signal A or B when present alone always preceded a resource. However, when A and B appeared together a resource never followed. This is a nonlinear classification problem known as the XOR problem (Haykin 1998). Circuits capable of solving a classification problem that remains unchanged over many generations are distinct from those circuits capable of associative learning *within a lifetime*. Consider this example—lifetime learning would be adaptive if, within the lifetime of a single bacterium, signal A (not B) predicted a resource, but, at other times within the lifetime, signal B (not A) predicted the same resource. If costly protein synthesis were required to exploit this resource, then a cell capable of learning the correct predictor out of many possible signals would be at a selective advantage because it could synthesize the proteins only after the correct predictor signal appeared.

To summarize Tagkopoulos *et al*'s important and distinct contribution, we clarify what they mean by ‘predictive behaviour’. Whereas metazoan nervous systems (and our proposed circuits) are capable of learning to predict temporal contingencies that vary within lifetimes, i.e. they are capable of learning conditioned stimuli, Tagkopoulus's mechanism depends on evolution and the fixity of the relationships between lifetimes, i.e. their mechanism is capable of *evolving* responses to unconditioned stimuli, but not of learning to predict a contingency within a lifetime. Their ‘predictive’ and ‘anticipatory’ behaviour is predictive and anticipatory in the sense that the evolutionary system causes salivation to ‘predict’ or ‘anticipate’ the presence of food after the smell of food.

Our work demonstrates that there exists sufficient variation in intra-cellular circuits to sustain Hebbian learning, the results of which can be epigenetically inherited. There is no doubt that epigenetic inheritance occurs in bacteria, single-celled fungi and protests (Jablonka & Lamb 2005). However, in nature, associative learning may not be observed in bacteria for any of the following reasons. The cost of maintaining associative learning machinery may be too high, and/or environmental affordances may be lacking (Rando & Verstrepen 2007). However, the circuits we propose may in future have medical applications, and therefore warrant a synthetic biological investigation. Synthetic biology was initially concerned with purely reactive behaviours (Endy 2005). Taking a significant step forward, recent work has dealt with state-dependent processing or ‘sequential logic’ gene circuits (Fritz *et al*. 2007). These circuits are capable of storing a binary memory vector representing the concentration of dimerizable proteins. Our work is similarly motivated, and adds the potential for Hebbian learning of such continuous vectors. We present designs of biochemical networks capable of associative learning, using GRNs and protein kinase signalling networks. We use mathematical models and simulations of the GRN, with biologically realistic parameters, to demonstrate not only that the system learns but also that it would be possible, in principle, to construct such a system in bacteria.

## 2. Gene regulatory network implementation

The genetic circuit shown in part of figure 1*b* is in two halves, by analogy with the classical Hebbian neural circuit in figure 1*a*. One-half (on the left in the diagram) represents the known response to the unconditioned stimulus, and the other (on the right) the response to the conditioned stimulus that is to be learned. In practice, circuits could consist of more than two unconditioned stimuli, in which case the circuit could learn to associate any one of these unconditioned stimuli with a conditioned stimulus. Two stimuli are the minimum number required to demonstrate the principle of Hebbian learning.

The two halves of the circuit are structurally identical, with one exception: there is a non-zero basal concentration of the ‘weight’ molecule corresponding to the unconditioned stimulus (denoted *w*_1_). When the corresponding input molecule *u*_1_ enters the cell, it binds with the repressor molecule *r*_1_, leading to loss of repression. Together with the background supply of *w*_1_, an activator of gene expression, this has the effect of producing the output response molecule *p*. Moreover, the generation of *p* then feeds back and allows further production of the *w*_1_ molecule. This is a molecular implementation of Hebbian learning in which pairing of output activity and input activity strengthens the coupling between the two (Hebb 1949; Gerstner & Kistler 2002). The use of the repressor in the genetic circuit (which does not appear in the neural implementation) is simply because the input molecule will be small (so as to quickly cross the cell membrane) and would not typically be a direct activator of gene expression.

In the other half of the circuit, however, there is no background level of the *w*_2_ molecule. This means that the corresponding input molecule, *u*_2_ (corresponding approximately to the bell in Pavlov's experiment), while binding to the repressor *r*_2_, is insufficient to generate the output response *p*. However, presenting the two inputs *u*_1_ and *u*_2_ simultaneously (the ‘smell’ and the ‘bell’) will produce sufficient quantities of *p* so that the molecule *w*_2_ will be produced in abundance. Moreover, a relatively slow decay rate for *w*_2_ will then ensure that, for some subsequent time period, the circuit can produce *p* in response to *u*_2_ alone. The circuit has learned to associate the two inputs together, through a (temporary) increase in the concentration of the *w*_2_ molecule.

The equations governing the circuit are

(2.1) $$\frac{\text{d}p}{\text{d}t}=\sum_{j=1}^{N}\left[v_{p}\left(\frac{w_{j}^{4}}{K_{w}^{4}+w_{j}^{4}}\right)\left(1-\frac{r_{j}^{2}}{K_{r}^{2}+r_{j}^{2}}\right)\right]-\delta_{p}p,$$

(2.2) $$\frac{\text{d}w_{j}}{\text{d}t}=v_{w}\left(\frac{p^{2}}{K_{p}^{2}+p^{2}}\right)\left(1-\frac{r_{j}^{2}}{K_{r}^{2}+r_{j}^{2}}\right)-\delta_{w}w_{j}+\epsilon_{1}.$$

For the two stimulus case in figure 1, *N*=2. Notice that the equations are symmetrical in *w*_1_ and *w*_2_, with the exception of the basal rate *ϵ*_1_ that is non-zero for weights representing unconditioned stimuli, i.e. *w*_1_. We assume all binding sites bind dimers: this creates a relatively sharp switching behaviour. In addition, the binding of the weight molecules *w*_1_ and *w*_2_ as promoters for *p* require two dimers to be bound cooperatively (as indicated by the Hill coefficient of four). The purpose of this (along with the relatively high Michaelis constant *K*_w_) is to create a small delay in the start of the *p* response. This is necessary to create a clear separation between the ‘on’ and ‘off’ responses to *u*_2_, so that the response to the conditioned stimulus will not accidentally be switched on by a few stray molecules. This means that the circuit will be robust to small fluctuations in the numbers of protein molecules. We use a relatively high production and degradation rate for *p* to give a strong response to the presence and absence of the input molecules. The weight molecules, on the other hand, degrade slowly. The model that we describe is deterministic and thus describes the average behaviour of a population of bacterial cells. It would be possible to formulate the model in a stochastic form that could capture the fluctuations in the numbers of proteins between individual cells in the population (Swain *et al*. 2002). While such a model would have the value of added realism, the structural properties of the system that give rise to associative learning are more clearly expounded and analysed with the small number of differential equations that we present.

We simulate this system by numerically integrating the above equations, for various input sequences. The parameter values we use are shown in table 1. It should be noted, however, that the behaviour of the circuit is robust to variations (of up to 25%) in these values.

It is assumed that there is a fast reversible reaction between the input molecules and the repressors. This means we can approximate the level of a repressor *r* as a function of the corresponding input *u* as

(2.3) $$r(t)=\frac{R}{1+ku(t)},$$

where *R* is the total amount of *r* in the system and *k* is the ratio of the forward to backward reactions.

## 3. Model behaviour

The results of a typical simulation are shown in figure 2. A series of input spikes of both *u*_1_ and *u*_2_ are presented. The spikes are of concentration 100 nM, and last for 20 min. (A duration of at least 5 min is necessary to get a response.) The system starts in the steady state with no inputs (this is [*p*]=0 nM, [*w*_1_]=500 nM, [*p*]=*0* nM, [*w*_1_]=500 nM, [*w*_2_]=0 nM). The first (unconditioned) input *u*_1_ is presented at *t*=2000 s and there is an immediate corresponding output spike of *p*, which almost attains the equilibrium level of 200 nM, and then swiftly disappears when the input spike finishes. A corresponding increase in the level of *w*_1_ can also be seen at this time. The presentation of a spike of *u*_2_ at *t*=6000 s produces no response at all. However, when spikes of *u*_1_ and *u*_2_ are presented simultaneously at *t*=10 000 s, we get a large (double) output response. This is because copies of *w*_2_ are now being rapidly produced, which enables *p* to be produced in response to both inputs. More importantly, the level of *w*_2_ is so large that, even when the inputs are switched off, it remains in the system for a considerable time. This means that when *u*_2_ is presented alone at *t*=40 000 s (that is, over 8 hours after the simultaneous presentation of *u*_1_ and *u*_2_), the circuit can still respond and produce a corresponding *p* spike.

A mathematical analysis of the equilibrium states of the system, when *u*_2_ is presented (but *u*_1_ is not), reveals a bistable switch. One stable state, the off state, has [*p*]=0.005 nM and [*w*_2_]=0.0001 nM. The other stable state, the on state, has [*p*]=200 nM and [*w*_2_]=9412 nM. They are separated by an unstable intermediate state with [*p*]=2 nM and [*w*_2_]=15.82 nM. The level of *w*_2_ dominates the switch from one state to the other. If this is below the switching threshold, then there is no response by the circuit. Levels of *w*_2_ above the threshold are able to produce a significant *p* response. In the simulation shown, *w*_2_ has a concentration of 56 nM at the time of the final *u*_2_ spike; more than enough to generate a full *p* spike. With the given set of parameters, the conditioned response continues for approximately 10.5 hours, by which time [*w*_2_]=22.8 nM. Any longer and it is lost, as the level of *w*_2_ goes below the threshold. In practice, the cell would have divided before this time, with the possibility that the offspring can inherit the learned association from the parent (depending on how much *w*_2_ is diluted by the split).

It should be noted that, although the increase in the *w*_1_ and *w*_2_ molecules is dramatic, it would take a long time (over 8 hours with the input molecules present) to get close to the equilibrium level of 9412 nM. Again, the cell would have divided before this occurs.

## 4. Experimental design

A preliminary design for the implementation of one-half of this genetic circuit is shown in figure 1*c*. This design makes use of two extrachromosomal plasmid replicons, within an *Escherichia coli* host, to maximize modularity and facilitate ‘mix and match’ mutagenesis. The plasmid replicons employed would need to be compatible and carry different antibiotic resistance genes. There are many possible combinations of activator and repressor proteins which could be used. An example set-up is as follows.

The first plasmid, pW1, contains the genes for the repressor (TetR) and for the weight molecule (FNR). The input stimulation would be sub-antibiotic concentrations of tetracycline, which alleviates repression by TetR and thereby induces FNR expression. The second plasmid, pRep1, will contain the gene for the output (orfP). This synthetic gene will encode a fused combination of cI activator protein, which activates the expression of the weight gene FNR, and a GFP reporter protein that causes fluorescence, thus enabling the output to be measured empirically. This gene is activated by FNR and repressed by TetR.

The output gene for *p* will also have a second promoter upstream, pBAD, which may be induced by arabinose, which activates the pBAD promoter via AraC protein and switches on expression of *p*. This is useful as a manual ‘switch’ for training and testing purposes.

The second half of the circuit would have the same structure, but make use of the *lac* repressor (to be modulated by the input) and CRP (cAMP receptor protein) as the second weight *w*_2_. The only structural difference between the two halves is in the basal concentration of the ‘weights’. This can be achieved by having either a leaky promoter for *w*_1_ or a separate gene on pW1 for generating the required background levels.

Extension of the circuit to *N* inputs requires a systematic approach constructing plasmid modules that can rapidly be structured into any desired topology of interactions, such as described in the framework of ‘programmable cells’ (Kobayashi *et al*. 2004). With our modular design, one can add more new inputs or associations—by adding extra copies of the P gene (i.e. on plasmids pRep2, pRep3 or more probably on the chromosome) with different promoters that have the required binding sites for new circuits. Of course this might be limited by finding new pairs of regulators with the desired characteristics, but sooner or later these may be available to order as part of coordinated approaches such as the BioBricks project (Knight 2003).

It should be noted that the relevant characteristics of the circuit components can often be readily adjusted by mutagenesis in order to attain an optimal response. In addition, further adaptations may be directed by artificial evolution *in vivo*, see electronic supplementary material for descriptions of *in silico* and *in vivo* evolution of Hebbian learning circuits.

## 5. Phosphorylation cycle implementation

An alternative method for implementing the same associative learning process within a single cell is to make use of phosphorylation cycles. The basic design is shown in figure 1*d*. The design requires proteins that can be single- and double-phosphorylated (such as MAPK protein kinases). The output response is given by the double-phosphorylated *p* protein. This then promotes the phosphorylation of the weight proteins, which are then further phosphorylated by the input molecules. The weights and inputs are both required to produce the outputs. All rates are assumed to be fast, except the decay of the double- to single-phosphorylated *w* proteins. This slow decay again preserves the conditioned learning response. To ensure sensitivity to the input *u*_1_, there needs to be a background rate for phosphorylating the *w*_1_ protein.

This implementation of the circuit is interesting as it illustrates an alternative mechanism by which associative learning could occur naturally in individual cells, see electronic supplementary material for a simple model of the MAPK system. Moreover, one would expect the response rates using phophorylation cycles to be much faster than for a GRN implementation. However, for experimental purposes, GRNs on plasmids are easier to synthesize and have the added advantage that they can be further evolved in the laboratory to optimize the behaviour.

We note that combinations of gene circuits and protein–protein interactions may be used to implement sequential logic operations as described by Fritz *et al*. (2007). Similar techniques could be used to modify our Hebbian circuits; see electronic supplementary material. The construction of novel GRNs is central to the synthetic biology project and there are many published examples of circuits designed to confer novel desired functions on micro-organisms, some of which have been constructed and tested (Elowitz & Leibler 2000; Kobayashi *et al*. 2004). ‘Toggle-switch’ circuits have been synthesized, conferring a form of memory on bacteria—a prolonged response to transient signals (Gardner *et al*. 2000).

## 6. Conclusions

We have presented a mathematical model of a GRN that is capable of associating two (or more) input signals in a Hebbian fashion. A design for implementing this circuit on plasmids in *E. coli* has also been described, along with an alternative implementation using phosphorylation cycles. The circuit could be tuned by directed evolution. Moreover, one would also expect to see inheritance of the learned association if the cell divides sufficiently soon after the learning takes place. See Jablonka & Lamb (2005) for a discussion of other mechanisms of epigenetic inheritance.

An important criticism is that the system is capable of learning only associations between *N* pre-defined dimensions of conditional stimuli, *u*_*j*_, and an unconditioned stimulus. We can give three responses.

Firstly, the inducer *u*_*j*_, which we have loosely been talking of as the stimulus, can without loss of generality be a downstream-regulated component or a second messenger that is activated by a wide range of possible signal transduction cascades. Any stimulus that activates this cascade can be associated with the unconditioned stimulus. The signal transduction cascade that produces *u*_*j*_ defines a *perceptual class* of stimuli that can be associated. Although it may be asking a lot of a bacterium that ‘a completely new’ association be learned, what would this mean? We argue it would mean that the bacterium was capable of creating a new perceptual class of stimuli to activate a given *u*_*j*_ molecule. One possible mechanism that would be capable of generating a novel perceptual class, within the lifetime of a cell, is intra-cellular natural selection, a process proposed by Wills (2001). If there is a source of sequence variation and heredity in an autocatalytic element of the signal transduction cascade, then natural selection can act between these elements, within the lifetime of a cell. Less ambitious is to propose that a random search process could establish novel perceptual categories. An even simpler mechanism would be if low sequence-fidelity transcription or translation produced variations in the selectivity and sensitivity of the components of a signal transduction system *within the lifetime* of a cell. We argue that signal transduction-based classifiers are analogous to perceptual areas in nervous systems that are responsible for defining classes of event by using topographic maps that represent increasingly abstract entities or Gestalts (Gibson 1986), rather than signalling atomic stimulus events. Much of the flexibility of an associative learning system depends on this transduction/classification process.

Our second response is that no learning mechanism is completely general, i.e. capable of associating any conditioned stimulus with any unconditioned stimulus. No neural network has the capacity to learn associations between *any* two inputs within the lifetime of the organism. The opposite belief was once held by the behaviourists who claimed that any reinforcer could strengthen any response (R) in the presence of any stimulus (S), provided that the animal could sense the stimulus and that a response was within its motor capabilities (Watson 1930; Skinner 1976). R and S were considered arbitrary, in effect symbolic items to be manipulated by this general learning system. A classic publication in psychology is ‘The misbehavior of organisms’ that describes experiments showing evolutionary constraints on what can and cannot be conditioned in mammals (Breland & Breland 1961). Later, Shettleworth denied that general laws of learning existed, claiming that the learning abilities of different species were specifically adapted to ecological constraints, i.e. learning was contingent on particular stimuli and responses and not independent of them (Shettleworth 1998). Further attempts to rectify a general theory of learning were made by Dickinson who claimed that particular features of ‘causes and effects’ in the ecological theory of an animal's lineage determined the properties of an animal's learning mechanism (Dickinson 1980).

Thirdly, the lack of flexibility serves to highlight that, although associative learning is possible in a single cell, nervous systems allow greater capacity for constructing novel pathways between arbitrary stimuli, because, instead of the network being defined by unchanging nucleotide and amino acid sequences, the spatial location of a synapse defines the pathway and ‘meaning’ of a signal. Nevertheless, one should point out that spatial localization in single cells does contribute to reaction specificity, and so could be another basis for associative learning (Harold 2005).

Finally, it is interesting to speculate about the potential applications of such molecular circuits. One idea is to use them as intelligent biomarkers for reporting on existing associations between cellular components. To do this, one constructs *N* plasmids as described above, whereupon Hebbian learning is used to train the resulting gene regulatory perceptron to classify the observed input vector. Secondly, a therapeutic bacterial system might adaptively tailor the anticipatory release of a drug to predict antecedents to a toxin. The genetic engineering of ‘remote-controlled’ bacteria to secrete drugs is already underway (Rao *et al*. 2005; Loessner *et al*. 2007). The circuit described provides a potential basis for such systems to learn.

## Figures and Tables

**Figure 1:**
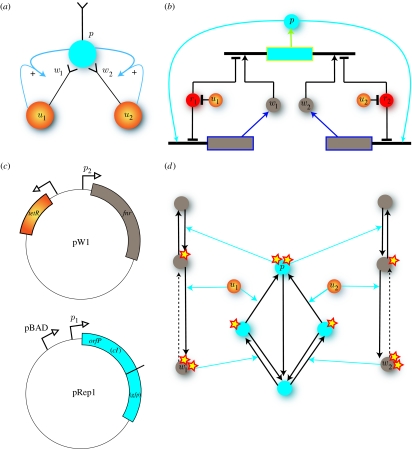
(*a*) The neural network implementation of Hebbian learning for two inputs *u*_1_ and *u*_2_. The orange circles represent pre-synaptic neurons that project onto a single post-synaptic neuron (blue). The simultaneous firing of the input neurons causes the synaptic weights *w*_1_ and *w*_2_ to increase, reinforcing their association. The blue curved lines show how this Hebbian positive feedback works, e.g. the weight *w*_1_ increases as a product of the output firing rate *p* and the input firing rate *u*_1_. (*b*) The equivalent gene circuit implementation using three genes is shown. The two input molecules (enhancers) are shown as orange circles, *u*_1_ and *u*_2_. They bind to the repressors (red circles) *r*_1_ and *r*_2_ and this results in activation of transcription of *w*_1_ and *w*_2_ molecules (in conjunction with transcription factor *p*) and activation of transcription of the *p* molecule (in conjunction with *w*_1_ and *w*_2_). To correspond to (*a*), the output molecule *p* is shown in blue. (*c*) Plasmid structures that could implement one half of the circuit. The first plasmid contains *fnr* and *tetR*. The second plasmid contains *orfP* (*cI*) and *gfp*; see text for details. (*d*) Alternative implementation using phosphorylation cycles. The inputs are again shown as orange circles *u*_1_ and *u*_2_; here they represent kinases that do one of two phosphorylation steps on the weight molecules *w*_1_ and *w*_2_ again shown in grey. The first phosphorylation step is done by a double phosphorylated output molecule *p*. Phosphorylation state is represented as yellow stars; one star means single phosphorylated, and two stars means double phosphorylated. Reversible and irreversible reactions are shown. The dotted arrow from $w_{1}^{**}$ to $w_{1}^{*}$ and from $w_{2}^{**}$ to $w_{2}^{*}$ indicates that this reaction is slow, i.e. that memory persists in the form of double phosphorylated $w_{1}^{**}$.

**Figure 2:**
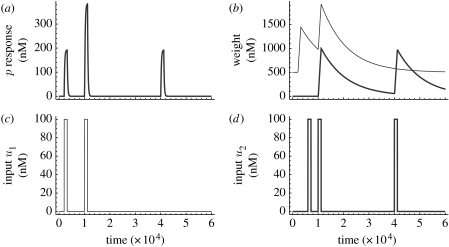
(*a*) The concentration of *p*. (*b*) The ‘weight’ molecule concentrations, *w*_1_ (thin line) and *w*_2_ (thick line). (*c*) The (unconditioned) *u*_1_ input concentration. (*d*) The (conditioned) *u*_2_ input concentration. The circuit responds by producing an output *p* (see the first peak of (*a*)) to input *u*_1_ at 2000 s (see the first peak of (*c*)). This demonstrates that *u*_1_ is the unconditioned stimulus. The circuit does not respond to the conditioned stimulus input *u*_2_ (see the first peak in (*d*)) at 6000 s when it is presented alone before pairing. Both *u*_1_ and *u*_2_ are presented paired together at time 10 000 s, resulting in an output of *p* (see the second peaks in (*c*) and (*d*) and the corresponding output *p* in (*a*)), and an increase in *w*_2_ from baseline to approximately 1000 nM in concentration (see (*b*)). The circuit then responds to *u*_2_ occurring 30 000 s later (third peak in (*d*)) by expressing *p* (see the third peak in (*a*)), where, before pairing, it had not responded to *u*_2_ at all (see absence of peak in (*a*) at 6000 s when *u*_2_ is presented for the first time). This demonstrates associative learning has indeed taken place.

**Table 1:** Parameter values used in circuit simulations. (Concentrations are in nmol per litre, and time is in seconds. These values are based on the results of using the SBMLevolver tool, a program that estimates the values of parameters for molecular circuits with known behaviour (Lenser *et al*. 2007).)

*v*_*p*_	1.0	production rate for *p*
*v*_*w*_	1.0	production rate for *w*
*δ*_*p*_	0.005	degradation rate for *p*
*δ*_*w*_	0.0001	degradation rate for *w*
*ϵ*	0.05	basal production rate for *w*_1_, unconditioned stimulus
*K*_*w*_	50	Michaelis constant for *w* promoting *p*
*K*_*r*_	0.05	Michaelis constant for *r* repressing *p* and *w*
*K*_*p*_	50	Michaelis constant for *p* promoting *w*
*R*	10	total level of repressors *r*
*k*	10	equilibrium ratio for reaction *r*+*r*⇔*ur*

## Supplementary Material

Supplementary Material

Mathematica File

Powerpoint animation
