# Supplementary material for: Molecular circuits for associative learning in single-celled organisms
Source: J R Soc Interface. 2008 Oct 3;6(34):463–9. doi: 10.1098/rsif.2008.0344 (PMC2582189; doi:10.1098/rsif.2008.0344)
Supplement: Supplementary file 2 [file rsif20080344s02.doc]

**Molecular circuits for associative learning in single-celled organisms**

Chrisantha T. Fernando^1,5^, Anthony M.L. Liekens^2^, Lewis Bingle^1^_,_ Christian Beck^4^, Thorsten Lenser^4^, Dov Stekel^1^, Jon Rowe^3^

*^1^ Systems Biology Centre, University of Birmingham, Birmingham, B15 2TT, UK*

*^2^TU/e Techniche Universiteit Eindhoven, the Netherlands*

*^3^ School of Computer Science, University of Birmingham, Birmingham, B15 2TT, UK*

*^4^ Bio Systems Analysis Group, Friedrich Schiller University Jena, Germany*

*^5^MRC National Institute for Medical Research, Mill Hill, London, London NW7 1AA, UK*

**Supplementary Material**

**1. Protein-Protein Interactions and Modifications to Hebbian Learning**

**Figure S1.** Additional synthetic components improve the robustness of the Hebbian learning circuits. See text.

As described in Fritz et al (2007), protein-protein interactions can be designed to modify synthetic gene circuits. Hebbian learning is known to be inherently unstable, and various techniques are known in neuroscience to deal with runaway positive feedback. In the main paper we have used simple transcription factor decay to prevent runaway positive feedback. However, figure S1 shows a range of optional extras for a synthetic Hebbian circuit. (A) Autocatalytic *p* and *w_j_* transcription factors may permit the maintenance of memory over more generations. The gating of this positive auto-regulation by a growth-phase dependent sigma factor ensures that weights and outputs do not increase during stationary phase. (B) Subtractive and (C) multiplicative normalization of weights^8^ can be implemented by heterodimer (*p + u_j_*) and homodimer (*p + p*) mediated proteolysis of the transcription factor *w_j_.* The later allows competition for limited ‘weight resources’. (C) The BCM rule (Dayan & Abbott, 2001) can be implemented by using an extra molecule *m* to represent the sliding average of *p* or *u_j_,* which then combines with *u_j_* ­and *p* to degrade weights.

1.1 **Pre-synaptic rule (not shown in Figure S1)**

$\frac{\text{dw}_{\text{ij}}}{\text{dt}}=h_{o}(v_{i}-v_{q})u_{j}$	…S1

can be implemented simply by adding a constant decay to the weight equal to $-h_{o}v_{q}u_{j}$­, that is zeroth order w.r.t. weight. This can be done if u_j_ is a saturated enzyme that degrades w at a constant rate, or if u linearly activates an enzyme that does this job. It should be noted that the pre-synaptic rule is unstable. For $v_{i}>v_{q}$then weights grow without bound, whereas for $v_{i}<v_{q}$ weights decay to zero. The post-synaptic rule is a minor modification, with decay of weights in proportion to $-h_{o}u_{q}v_{i}$ rather than $-h_{o}v_{q}u_{j}$.

1.2. **Saturating weight dependence rule (Figure S1B).**

$\frac{\text{dw}_{\text{ij}}}{\text{dt}}=h_{o}(w^{\text{max}}-w_{\text{ij}})v_{i}u_{j}$	…S2

works by reducing the increase in weights as weights go to w^max^, and actually decreasing weights if the weight goes above w^max^. Here the *w_­ij_* transcription factor is destroyed at rate $v_{i}u_{j}$ in the first order. This can be implemented if a heterodimer $v_{i}u_{j}$ acts to degrade w­_ij_ directly.

1.3. **Oja’s rule (Figure S1C)**

$\frac{\text{dw}_{\text{ij}}}{\text{dt}}=h_{o}v_{i}u_{j}-{\alpha v}_{i}^{2}w_{\text{ij}}$	…S3

has a very similar form, except that here the homodimer v^2^ is responsible for the first order decay of w_ij_. See that S3 differs from S2 in that the decay is transmitted to all weights via *v* even if that particular weight did not cause the production of *v*, thus implementing competition

1.4. **Weight consolidation rule** **(not shown in figure S1)**

$\frac{\text{dw}_{\text{ij}}}{\text{dt}}=h_{o}v_{i}u_{j}-{\alpha w}_{\text{ij}}(1-w_{\text{ij}})(w_{q}-w_{\text{ij}})$	…S4

($0<w_{q}<1$ and $\alpha>0$) can be implemented by introducing a autocatalytic promotor for w­_ij_ with Hill-coefficient for w_ij_ binding of n = 2. The strength of the autocatalytic promotor of w­_ij_ is equal to $\alpha(1-w_{q})$. At the same time there must be decay of w_ij_ at a first order constant rate $-{\alpha w}_{q}$ *and* a decay of w­_ij_ at a third order rate $\alpha$! This is a complex process to implement, but it is conceivable that such processes could evolve if selection existed for consolidation of transcription factor cytoplasmic memory.

1.5. **BCM rule (Figure S1D)**

The rule is described by:

$\frac{\text{dw}_{\text{ij}}}{\text{dt}}=h_{o}f(v_{i}-\left\langle v_{i} \right\rangle)u_{j}$	…S5

$f$ is a zero crossing function with positive slope for example v_i_(v_i_- $\left\langle v_{i} \right\rangle$). $\left\langle v_{i} \right\rangle$ is a short-term average of output activity and is given for example by

$\frac{d\left\langle v_{i} \right\rangle}{\text{dt}}=n_{v}(v_{i}^{2}-\left\langle v_{i} \right\rangle)$	…S6

Let us first implement the sliding threshold 10.1. This is simply a TF that is activated by the output ­*v­* and decays at constant rate n_v_. The production of w_ij_ is at rate $h_{o}$v_i_^2^u_j_ and w_ij_ decays at rate $h_{o}$v$\left\langle v_{i} \right\rangle$u_j_. Here, three species must come together to destroy w_ij_, v, $\left\langle v_{i} \right\rangle$, and u­_j_. This rate must be 0^th^ order, i.e. independent of the concentration of w_ij_. $\left\langle v_{i} \right\rangle$ can be implemented as another molecule *m* (Figure S1D), that is activated by *v* with Hill Coefficient = 2, and decays at 1^st^ order with the same time constant as its production.

**2. Evolution of Circuits *In Silico***

For the *In silico* optimization we expanded the SBMLevolver of Thorsten Lenser, which implements a new 2-layer evolutionary algorithm. The model in the main paper is a subsequent version optimized by hand, but based on the evolved parameters below. The upper level evolves the structure of the network using genetic programming while the lower level uses a evolution strategy to optimize only the parameter of the network. For the upper level we had a (10+10)-elitism strategy over 500 generations, that means in each generation we selected, based on the fitness rank, the 10 best individuals as parents for the next generation and created 10 new offspring. As we have used the network in figure S3 as the starting network and don’t want to mutate this, the offspring are just a copy of the parents. In the lower level we had a (2+2)-elitism strategy over 10 generations.

After all we received a network with the output you can see in figure 4 in the original paper.

The values of the signals - remember they are repressor - is 200 if they are inactive and 105 if they are active.

For the parameter of the network remember the equations:

$\frac{\text{dw}_{j}}{\text{dt}}=h_{j}\left( \frac{p^{n_{j1}}}{K_{p}+p^{n_{j1}}} \right)\left( 1-\frac{r_{j}^{n_{j2}}}{K_{u}+r_{j}^{n_{j2}}} \right)-d_{j}w_{j}$	…S7

and

$\frac{\text{dp}}{\text{dt}}=\sum\left[ h_{j}\left( \frac{w_{j^{n_{j1}}}}{K_{w_{j}}+w_{j^{n_{j1}}}} \right)\left( 1-\frac{r_{j}^{n_{j2}}}{K_{u_{j}}+r_{j}^{n_{j2}}} \right) \right]-{Nd}_{p}p$	…S8

|  | $h$ | $n_{1}$ | $n_{2}$ | $K_{1}$ | $K_{2}$ | $d$ |
| --- | --- | --- | --- | --- | --- | --- |
| $\frac{\text{dw}_{1}}{\text{dt}}$ | 29.911 | 1.063 | 4.725 | $K_{p}=\text{4995}\text{.}\text{284}$ | $K_{U_{1}}=\text{215}\text{.}\text{629}$ | 0.00010 |
| $\frac{\text{dw}_{2}}{\text{dt}}$ | 23.565 | 1.045 | 4.717 | $K_{p}=\text{3006}\text{.}\text{691}$ | $K_{U_{2}}=\text{203}\text{.}\text{137}$ | 0.00012 |
| $\frac{\text{dp}}{\text{dt}}$ | 40.835 | 4.661 | 2.933 | $K_{w_{1}}=\text{10015}\text{.}\text{536}$ | $K_{U_{1}}=\text{198}\text{.}\text{309}$ | 0.28223 |
|  | 30.337 | 4.135 | 2.315 | $K_{w_{2}}=\text{10009}\text{.}\text{036}$ | $K_{U_{2}}=\text{2014}\text{.}\text{580}$ |  |

**Table S1.** The parameter of the evolved network. The concentrations at the beginning are 3.942(w1), 0.021 (w2) and 0.0002 (p)

To find this learning network we had to define a new fitness function. To make the explanation of the function easier we have to make a definition. We split one experiment into 9 sections:

|  | Activation of signals | Timesteps |
| --- | --- | --- |
| 1 | At the beginning, no signals. | 1000 |
| 2 | First activation of signal 1 (U1). | 200 |
| 3 | Back to no activation | 2000 |
| 4 | First activation of signal 2 (U2). | 200 |
| 5 | Again no activation | 1500 |
| 6 | The pairing in the test case, so both signals are active (U1+U2). In the control case only signal 2 is active once more, no pairing | 200 |
| 7 | Memorise phase, no signals active | 7000 |
| 8 | Test phase, signal 2 active for the last time | 200 |
| 9 | At the end, no signals | 1000 |

So we define $T_{j}(O)$ as the value of protein O at the last timestep in section j in the test case, analogous $C_{j}(O)$ as value of O in section j in the control case.

Then our fitness function looks like this:

$$\begin{aligned} \frac{\text{fit}=c_{\text{const}}+T_{7}(P)overbracealignl\overset{\text{signal is active}}{\overbrace{\begin{aligned} \text{min}\text{.}\text{ P if no} \end{aligned}}}+\text{100}*T_{4}(P)overbracealignl\overset{\text{to signal 2}}{\overbrace{\begin{aligned} \text{min}\text{.}\text{ response} \end{aligned}}}}{(T_{8}(P)-T_{7}(P))underbracealignl\underset{\text{test case}}{\underbrace{\begin{aligned} \text{maximise learning in} \end{aligned}}}\text{Set to 0, if < 0}} \end{aligned}+(T_{8}(P)-C_{8}(P))underbracealignl\underset{\text{control case}}{\underbrace{\begin{aligned} \text{minimise learning in} \end{aligned}}}\text{Set to 0, if < 0}+(T_{2}(P)-T_{4}(P))underbracealignl\underset{\text{to U2 compared to U1 }}{\underbrace{\begin{aligned} \text{min}\text{.}\text{ output as response} \end{aligned}}}\text{Set to 0, if < 0}+(T_{2}(P)-T_{1}(P))underbracealignl\underset{\text{to signal 1}}{\underbrace{\begin{aligned} \text{max}\text{.}\text{ response} \end{aligned}}}\text{Set to 0, if < 0}+\underset{\text{max}\text{.}\text{ learning}}{\underbrace{T_{8}(P)}}+\left| C_{7}(P)-C_{8}(P) \right|underbracealignl\underset{\text{in control case}}{\underbrace{\begin{aligned} \text{minimize learning} \end{aligned}}}+\text{100}*\left| T_{8}(P)-T_{2}(P) \right|underbracealignl\underset{\text{without and signal 2 with learning}}{\underbrace{\begin{aligned} \text{forces an equal outputfor signal 1} \end{aligned}}}+\text{100}*\left| T_{1}(P)-T_{7}(P) \right|underbracealignl\underset{\text{artefact of non decay of P}}{\underbrace{\begin{aligned} \text{makes sure, learning is not an} \end{aligned}}}+\underset{\text{punishments}}{\underbrace{a+b+c+d+e+f}}$$

As the SBMLevolver tries to find the smallest fitness possible, it will reduce all added parts and terms over the slash and increase the values under the slash. The first 4 punishments have no influence in the learning of the network, they should lead to a reasonable network only. So we used the following:

a ... If one term in the main function goes to a negative number set this term to 0 and add 1000
b ... Add a punishment of 1000 if W_1_ or W_2_ still growing in section 9. If so, it will never reach to state of the beginning again.
c ... Add the differences of the concentrations of P, W_1_ and W_2_ between the first and the last timestep in section 1. This leads to a steady state at the beginning.
d ... Add 1000 if any of the concentrations goes over the maximal concentration which is 200 in our case (the concentration of the signals if not active).

We also added two punishments, which have a influence in the learning of the fitness:

e ... Add 900 if $(T_{8}-T_{7})\text{<<}(T_{2}-T_{1})$ (gain after learning is much smaller than with signal 1) and
f ... add 1000 if learning in the test case is not more than a twice of learning in the control case.

This helps the evolutionary algorithm to move in the space, but will have no influence in the final result, because the premises will not be true.

The c_const_ in the main function is for regulation of the strength of the fraction. The higher c the weightier is the main function in relation to the punishments. In our case we used also 100000 for c_const_.

**3. Evolution of circuits *in vivo*.**

Figure S2 shows a proposed outline protocol for evolution of Hebbian learning circuits *in vivo­.* There are two phases of testing, the control and the test phase. In the control phase we do not pair the two stimuli and require no learning. In the test phase we pair two stimuli and require the learning of the association. A facs machine is used to select for no GFP expression, and high GFP expression respectively. Simulated evolution *in silico* was used to design an effective fitness function.

**Figure S2.** The overall protocol for artificial selection of plasmid gene circuits is shown. After circuits have been cloned in *E. coli* we will undertake high throughput selection using a classical conditioning task, to evolve bacterial colonies capable of robust conditioning. A FACS machine will be used to undertake selection using a 2-phase fitness function.

**4. Simple model of MAPK implementation**

The reactions describing the MAPK circuit are shown below:

with the corresponding differential equations:

…32*

**Figure S3.** U1Copy and U2Copy represent input chemicals. Initially W11 starts at 0.1, and V11 starts at 0. So that U1 initially activates the output, by U2 does not. After pairing of U1 and U2, output P11 is produced just with U2 input alone. This was not the case before pairing.

A classical conditioning experiment is shown in figure S3.

The Cellerator^TM^ file (Bruce Shapiro) for Mathematica is available on on-line, and was used to simulate this system. These equations represent mass action kinetics, although more complex models are possible. The basic organization of Hebbian learning can be appreciated. There are two positive feedback loops, coupled by a common output signal. Both output and input must be present for weight increase. Weights and outputs are both double phosphorylated protein kinases. The single phosphorylated and unphosphorylated forms are intermediates in the circuit, without any enzymatic activity of their own. The system implements weight decay because w_11_ and v_11_ is dephosphorylated slowly back to w_01_ and v_01­_ respectively.

**5. References**

Dayan P, Abbott LF (2001) Theoretical Neuroscience: Computational and Mathematical Modeling of Neural Systems. (The MIT Press)
